# Supplementary material for: The Anti-Dengue Virus Peptide DV2 Inhibits Zika Virus Both In Vitro and In Vivo
Source: Viruses. 2023 Mar 25;15(4):839. doi: 10.3390/v15040839 (PMC10143277; doi:10.3390/v15040839)
Supplement: Supplementary file 1 [file viruses-15-00839-s001.zip › viruses-2291403-supplementary.pdf]

Supplementary Materials:

Table S1. Prediction of non-covalent interactions between the ZIKV E protein and the synthetic DV2 peptide in the postfusion conformation according to the PLIP tool [20]. Residues in bold belong to domain I and in black belong to domain II.

Protein residue		Peptide residue		Distance (Å)
		Hydrophobic interactions		
Trp	167	Phe	429	3.05
Trp	167	Phe	429	2.93
Trp	167	Ile	432	3.86
Trp	167	Ile	432	3.47
Ile	171	Ile	432	2.94
Phe	195	Ala	446	3.59
Val	206	Ile	443	3.94
Leu	208	Ile	443	3.37
Ala	214	Leu	436	3.15
Ala	218	Ile	432	3.67
Ala	222	Leu	425	3.91
Leu	223	Leu	425	3.75
**Glu**	**270**	Phe	422	2.94
**Val**	**276**	Phe	422	3.61
**Trp**	**350**	Phe	422	3.03
**Arg**	**352**	Phe	422	3.12
		Hydrogen bonds		
Thr	45	Ala	447	2.93
Leu	219	Val	428	2.85
**His**	**351**	Phe	422	2.40
**Ser**	**353**	Gly	423	3.36
		Salt Bridges		
**His**	**348**	Trp	420	5.66
**His**	**348**	Trp	420	5.94

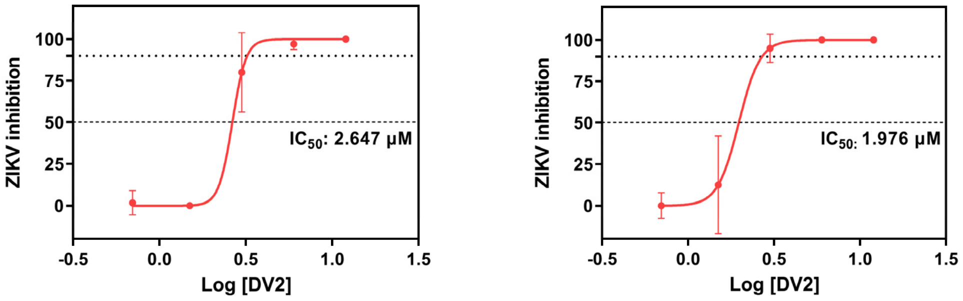

(A) (B)

	(A)	(B)
**LogIC50 (Std. Error)**	0.4228 (0.1421)	0.2957 (0.04430)
**HillSlope (Std. Error)**	11.08 (28.93)	7.050 (2.378)
**R^2**	0.9459	0.9386

Figure S1. Determination of DV2 peptide IC_{50} values against ZIKV through different techniques. IC_{50} was calculated using (A) flow cytometry and (B) plaque assays from a 2-fold dilution series. The IC_{50} curves were plotted using GraphPad Prism v. 9.0.0.
